# Effect of whole-body vibration on neuromuscular activation and explosive power of lower limb: A systematic review and meta-analysis

**DOI:** 10.1371/journal.pone.0278637

**Published:** 2022-12-06

**Authors:** Zhen Wang, Zhen Wei, Xiangming Li, Zhangqi Lai, Lin Wang

**Affiliations:** 1 School of Kinesiology, Shanghai University of Sport, Shanghai, China; 2 Guang Dong Youth Vocational College, Guangzhou, China; 3 The Third School of Clinical Medicine (School of Rehabilitation Medicine), Zhejiang Chinese Medical University, Hangzhou, Zhejiang, China; Sport Sciences School of Rio Maior - Politechnic Institute of Santarem, PORTUGAL

## Abstract

**Objective:**

The review aimed to investigate the effects of whole-body vibration (WBV) on neuromuscular activation and explosive power.

**Methods:**

Keywords related to whole-body vibration, neuromuscular activation and explosive power were used to search four databases (PubMed, Web of Science, Google Scholar and EBSCO-MEDLINE) for relevant studies published between January 2000 and August 2021. The methodology of the Preferred Reporting Items for Systematic Reviews and Meta-Analyses was used. The eligibility criteria for the meta-analysis were based on PICOST principles. Methodological assessment used the Cochrane scale. Heterogeneity and publication bias were assessed by I^2^ index and funnel plots, respectively. The WBV training cycle is a random effect model. Publication bias was also assessed based on funnel plots. This study was registered in PROSPERO (CRD42021279439).

**Results:**

A total of 156 participants data in 18 studies met the criteria and were included in the meta-analysis for quantitative synthesis. Results of the meta-analysis showed significant improvements in lower limb neuromuscular activation immediately after WBV compared with the baseline (SMD = 0.51; 95% CI: 0.26, 0.76; p<0.001), and no significant heterogeneity was observed (I^2^ = 38%, p = 0.07). In addition, the highest increase in lower limb explosive power was observed (SMD = 0.32; 95% CI: 0.11, 0.52; p = 0.002), and no significant heterogeneity (I^2^ = 0%, p = 0.80) was noted.

**Conclusions:**

WBV training could improve neuromuscular activation and explosive power of the lower limb. However, due to different vibration conditions, further research should be conducted to determine standardized protocols targeting performance improvement in athletes and healthy personnel experienced in training.

## 1 Introduction

The human body response to mechanical vibration has been widely studied since the middle of the 19th century [1]. In recent decades, vibration was recommended for use as a supplementary training in competitive sports, amateur sports and rehabilitation [2–6]. Many studies were conducted to investigate the influence of whole-body vibration (WBV) training on sports performance. Their findings showed the positive influence of WBV training on neuromuscular activation, muscle strength, power, movement speed, jump, flexibility and balance [7–14]. The comprehensive description of WBV currently used by athletes includes type of equipment, physics principles, frequency, amplitude, acceleration, muscle and tendon mechanics, and neuronal and physiological responses [6]. For athletes, neuromuscular activation and explosive power are one of the important indicators affecting sports performance; therefore, much attention has been paid to the study of neuromuscular activation and explosive power. Currently, studying the acute effects of precise WBV training protocol on neuromuscular activation and explosive power in athletes is particularly important.

In previous studies, WBV training could improve muscle activation [15–17]. A common mechanism for WBV-induced enhancement of muscle activity is tonic vibration reflex (TVR) resulting from muscle spindle and α-motor neuron activation [18, 19]. WBV acts on neuromuscular coupling and improves motor coordination [20, 21]. Neural enhancement at the spinal level may underlie WBV-induced improvement in coordination, but neuromuscular activation enhancement causes and mechanisms have received minimal attention [22]. Vibration activates muscle spindles, elicits alpha-motor neuron excitation and induces enhanced muscle activation [6]. Vibration training affects the neuromuscular system and may have positive acute and chronic effects on neuromuscular activation [23–26]. Cardinale and Bosco’s study found the potential function of vibration on neuromuscular activation can be performed with central and peripheral structures [27]. However, the review found that WBV has no or only minor additional effects on muscle strength, jump height and neuromuscular activation [28]. Many studies found that WBV training causes acute increases in muscle activation using surface electromyography (sEMG) during vibration exposure [29–34]. In the above literature study, sEMG was used to illustrate neuromuscular activation and muscle activation during vibration training.

Explosive power, the ability to produce high strength in the shortest time and known as the speed of strength development, is a quality possessed and optimized by elite athletes. Explosive power usually assessed as a vertical jump, is an important indicator of sports performance [20, 35–38]. Vertical jumping ability has been found highly correlated with weightlifting performance and sprint speed [20, 21]. In addition, explosive power is an essential component of important movements of team games, including basketball, volleyball and soccer [39–41]. Therefore, seeking various training strategies to develop explosive power is important. Many studies explore the application of mechanical vibration as a potential stimulus to increase muscle function and sports performance [42–44]. As mentioned above, WBV induces rapid stretch–shortening cycle that promotes muscle function through TVR, enhances muscle energy metabolism through vibration-induced muscle contraction and increases muscle perfusion rate, increases muscle temperature; this potential mechanism may exert beneficial effects on neuromuscular function on explosive power [39, 40]. Over the past decade, a number of studies investigated the effects of WBV on generating maximum voluntary muscle strength and explosive power in athletes. Inconsistent findings were found. For example, reviews reported no difference in muscle strength and explosive power improvement with WBV training [45].

A wide variety of exercise programs may influence outcomes. Systematic reviews of athletic performance and muscle activation and explosive power in athletes by WBV remain contradictory and controversy [9, 46, 47]. Therefore, further reviewing and analysing the effects of WBV training on neuromuscular activation and explosive power are necessary. Based on a preliminary analysis of the research literature, peak force, power and jump height were used to express explosive power and sEMG instead of neuromuscular activation to determine the acute effects of vibration on neuromuscular and explosive power.

## 2 Methods

This review followed the Preferred Reporting Items for Systematic Reviews and Meta-Analysis (PRISMA) statement guidelines [49]. The review protocol was registered with the International Prospective Register of Systematic Reviews (CRD42021279439).

### 2.1 Literature search and screening

The electronic databases of PubMed, Web of Science, Google Scholar and EBSCO-MEDLINE were searched online from 1 January 2000 to 31 August 2021. According to previous search results, there are almost no relevant research before year 2000. Therefore, the data of research search was selected at year 2000. The following search terms were applied in the database search: (vibration*[MeSH Terms] OR vibration*[All Fields]) AND ((neuromuscular activation*[MeSH Terms] OR neuromuscular activation*[All Fields]) OR (muscle activation*[MeSH Terms] OR muscle activation*[All Fields]) OR (explosive power*[MeSH Terms] OR explosive power*[All Fields]) OR (muscle power*[MeSH Terms] OR muscle power*[All Fields]) OR (muscle strength *[MeSH Terms] OR muscle strength *[All Fields])). A review of the references of the retrieved articles followed to ensure the comprehensiveness and accuracy of the relevant studies. The literature search was done with the assistance of librarians in Guangzhou Library. ZW and ZQL independently screened the title, abstract and full text, and for controversial articles, a third author checked them. Full search strategies for each database can be found in S1 Table.

### 2.2 Inclusion and exclusion criteria

The eligibility criteria for the meta-analysis were based on PICOST principles [48]: (1) healthy subjects; (2) acute WBV training intervention; (3) comparison before and after the experiment; (4) neuromuscular activation (sEMG, root mean square), explosive power (e.g. peak force, power and CMJ); (5) pre/post intervention or randomized controlled trial (RCT) studies; (6) total intervention time of 15–500 s.

**Fig 1:**
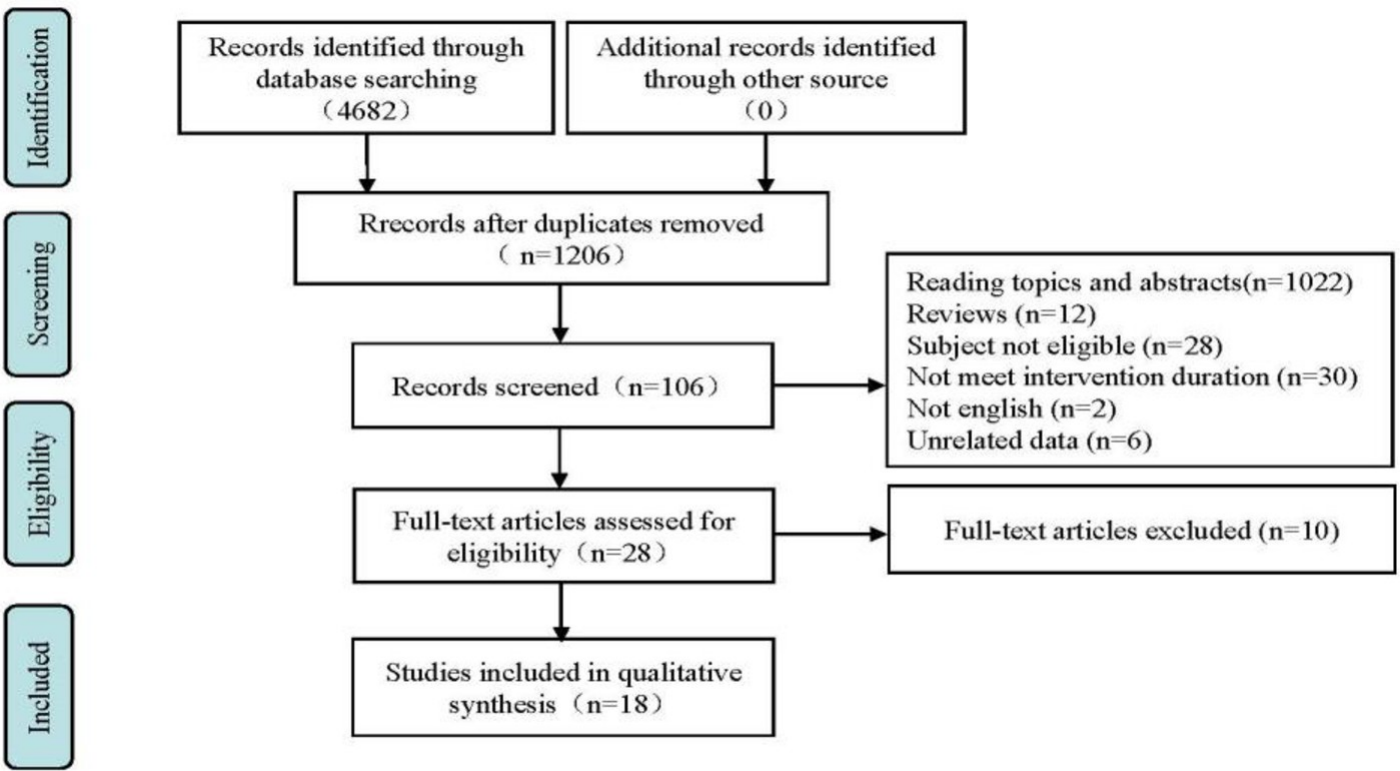
PRISMA flow chart of study selection process.

The exclusion criteria for this meta-analysis were as follows: (1) unpublished literature or conference abstracts; (2) animal experimental studies or review literature; (3) studies with only post-test and no pre-test; (4) repeated published studies or no full text; (5) literature in which data could not be extracted or combined. The meta-analysis was conducted according to PRISMA Statement Format, Study Literature Screening Flow Diagram [49] (Fig 1).

### 2.3 Data extraction process/data items

Data were extracted from the selected articles by one of the authors. The extracted data were checked by another author, and disagreements were resolved with a third. The following data were extracted for each selected article: (1) author, year; (2) subjects (e.g. age, gender and volleyball, football and soccer); (3) types and parameters (e.g. type of vibration, amplitude and frequency); (4) interventions (e.g. WBV, duration and position); (5) outcome characteristics (e.g. related to neuromuscular activation or explosive power).

### 2.4 Assessment of methodological quality

Each full-text article was assessed by two independent reviewers and scored using the Cochrane scale [50]. The Cochrane bias risk assessment tool mainly covers seven aspects: random sequence generation and allocation concealment, blinding of participants and personnel, blind evaluation of research outcome, completeness of outcome data, selective reporting and other bias. For each item, the judgment results of ‘low risk bias’, ‘high risk bias’ and ‘unclear’ were made according to the bias risk assessment criteria. Two assessors independently evaluated the quality of the included studies. In case of disagreement, a group discussion was held with a third expert to reach consensus.

### 2.5 Statistical analysis

To assess the effect of WBV on outcome measures, a meta-analysis compared the intervention groups before and after the experiment. Random effect models were used to calculate standardised mean differences and 95% confidence intervals (CIs) before and after the experiment. The I^2^ statistic was used to verify heterogeneity (χ^2^) between the included studies. The risk of publication bias was also assessed by using funnel plots. Statistical significance was set at p<0.05.

## 3 Results

### 3.1 Study selection

Fig 1 shows the PRISMA flow chart of the study selection. A total of 4682 references were screened. Duplicates were removed. The title and abstract of the remaining records were screened. A total of 106 articles remained and were assessed for eligibility, 28 articles were included in the final yield and the 18 remaining studies finally satisfied the eligible criteria in this meta-analysis.

### 3.2 Study characteristics

A total of 156 healthy participants were included in the analysis. The mean age in each article ranged from 9.22 years to 31 years. Subjects included active males [51], resistance training men [52], recreationally active [53–55], sport science students [56], physically active [57–60] and professional athletes [61–66]. The sample sizes of the included studies ranged from 9 to 60.

All 18 included studies analysed the acute effects of WBV on neuromuscular performance and explosive power. Of these 18 studies, 1 was pre/post-test design with no frequency or subject grouping [60], 7 did not state the randomization procedure [32, 55, 58, 62, 64–66] and 9 described the randomization method [16, 51–54, 56, 57, 59, 63]. The intervention time of the 18 studies varied: 15 s [32], 30 s [52, 54, 55, 59], 60 s [60–62], 120 s [64, 65], 150 s [51], 180 s [63], 240 s [16, 56] and 300 s [53, 57, 58, 66]. The changes in flexion angle were 5° [59], 10° [66], 30° [59, 66], 40° [32, 58], 60° [59], 90° [51, 55–57, 62, 65], 100° [16, 52, 54, 60, 63], 120° [61, 64, 65], 140° [53] and 150° [53]. The interventions varied in terms of vibration types, parameters, body posture and timeframes. The manifestation of neuromuscular activation was sEMG (biceps femoris, quadriceps femoris, gastrocnemius and soleus muscles), and the manifestation of explosive power was counter movement jump (CMJ), power and peak force. Table 1 shows the characteristics of the included studies.

**Table 1:** Description of the characteristics of the included studies.

Author, Year	Subjects	Types and Parameters	Interventions	Outcomes
Bosco et al., 2000 [60]	14 physically active males, mean age: 25.1±4.6, WBV	VP = vertical A = 4 mm F = 26 Hz a = 17 g	Subjects exposed to 10 times of WBV (a rest period lasting 6 min was allowed after five vibration sets) Duration: 60 s. Rest: 60 s. T: 60 s. Modality: toes on the vibration platform, wear gymnastic-type shoes Position: knee angle 100° flexion	NA: sEMG (vastus lateralis, rectus femoris) MP: CMJ, Mechanical W measurements
Hannah et al., 2011 [53]	14 recreationally active males, mean age: 23±3, WBV, CON, RCT	VP = NR A = 2, 4 mm F = 30–40–50 Hz a = NR	Participants completed 5×1 min bouts of exercise with 1 min of relaxed standing between each bout. Duration: 60 s. Rest: 60 s. T: 300 s. Modality: unilateral squat exercise, without shoes Position: knee angle 140°, 150° flexion	NA: sEMG (quadriceps) MP: NM
Borges et al., 2016 [58]	60 physically active women, mean age: 22.7±3.5, WBV, CON	VP = NR A = 4 mm F = 30–50 Hz a = NR	Subjects exposed to 10 times of WBV (10 sets of 30 seconds with a 60-second rest period between sets) Duration: 30 s. Rest: 30 s. T: 300 s. Modality: the non-dominant limb, barefoot, the upper limbs extended shoulder and the trunk kept upright Position: knee angle 40° flexion	NA: sEMG (vastus lateralis) MP: average power, peak torque
Ritzmann et al., 2013 [59]	18 physically fit students, mean age: 25±4, WBV, RCT	VP = vertical A = 2 mm F = 5–10–15–20–25–30 Hz a = NR	Duration: 10 s. Rest: 30 s. Modality: forefoot, normal stance, load variation (no load vs load equal to one-third of body weight) Position: knee angle 5°, 30°, 60° flexion	NA: sEMG (biceps femoris, quadriceps, soleus, tibialis anterior) MP: NM
Wu et al., 2021 [61]	20 man volleyball players, mean age: NR, WBV	VP = vertical A = 2 mm F = 30 Hz a = NR	Duration: 60 s. Rest: NR. Modality: NR, wore volleyball shoes Position: knee angle120° flexion	NA: NM MP: CMJ, 10 m sprinting, blocking agility test
Borges et al., 2017 [32]	40 physically active women, mean age: 22.9±2.8, WBV	VP = NR A = 2, 4 mm F = 25–50 Hz a = 2.5, 20 g	Duration: 15 s. Rest: 30 s. Modality: unilateral standing, the non-dominant limb, barefoot, the upper limbs extended shoulder and the trunk kept upright Position: knee angle 40° flexion	NA: sEMG (vastus lateralis) MP: NM
Cloak et al., 2016 [63]	44 women soccer players, mean age: 23.1±3.7, WBV, CON, RCT	VP = vertical A = 8 mm F = 40 Hz a = NR	Duration: 60 s. Rest: 60 s. T: 180 s. Modality: 100-degree squat, raise their heels as much as possible, the non-dominant limb Position: knee angle 100° flexion	NA: sEMG (quadriceps) MP: Peak isometric force.
Pedro et al., 2021 [55]	14 recreationally active students, mean age: 23.1±1.5, WBV	VP = NR A = 1.52, 4 mm F = 30–50 Hz a = 2.8, 5.4 g	Duration: 30 s. Rest:180 s. Modality: the bridge exercise, athletic shoes Position: knee angle 90° flexion	NA: sEMG (biceps femoris, semitendinosus, gluteus maximus) MP: NM
Dallas et al., 2015 [64]	18 divers, mean age: 17.9±2.4, WBV	VP = NR A = 2, 4 mm F = 30–50 Hz a = NR	Subjects performed a static squat at a knee angle of 120° and a dynamic squat at a tempo of 2 s up and 2 s down at a knee angle ranging from 120° to 180°. Duration: 30 s. Rest: 30 s. T: 120 s. Modality: static squat, dynamic squat, wore gymnastics shoes Position: knee angle 120°, 120°–180° flexion	NA: NM MP: CMJ, SJ flexibility-sit and reach test
Turner et al., 2011 [54]	12 recreationally active men, mean age: 31±8, WBV, RCT	VP = vertical A = 8 mm F = 30–40 Hz a = NR	Duration: 30 s. Rest: 180 s. Modality: half-squat position Position: knee angle 100° flexion	NA: NM MP: CMJ
Cormie et al., 2006 [52]	9 resistance training men, mean age: 19–23, WBV, RCT	VP = NR A = 2.5 mm F = 30 Hz a = NR	Duration: 30 s. Rest: NR. Modality: half-squat position Position: knee angle 100° flexion	NA: sEMG (vastus medialis, vastus lateralis, biceps femoris) MP: CMJ, IS
Chang et al., 2019 [62]	16 women fencing athletes, mean age: 19.6±1.1, WBV	VP = NR A = 2 mm F = 30 Hz a = NR	Duration: 60 s. Rest: NR. Modality: half-squat position Position: NR	NA: NM MP: CMJ, 10-meter sprint
Dallas et al., 2013 [65]	32 gymnasts, mean age: 9.2±1.3, WBV, CON	VP = vertical A = 2 mm F = 30 Hz a = NR	Duration: 30 s. Rest: 30 s. T: 120 s. Modality: standing on one leg, gymnastics shoes Position: knee angle 90°, 120° flexion	NA: NM MP: CMJ, SJ
Crow et al., 2012 [66]	22 football players, NR, mean age: 22.6±4.2, WBV, CON	VP = NR A = 6.4 mm F = 30 Hz a = NR	Duration: NR. Rest: NR. T: 300–420 s. Modality: static squat stance Position: knee angle 10°–30° flexion	NA: sEMG (gluteus maximus, gluteus medius) MP: CMJ, peak power
Colson et al., 2016 [57]	14 male physical education students, mean age: 23.1±0.9, WBV, RCT	VP = vertical A = 4 mm F = 30 Hz a = 7.2 g	Duration: 30 s. Rest: 30 s. T: 300 s. Modality: dynamic squatting, barefoot Position: knee angle 90° flexion	NA: NM MP: CMJ. flexibility-sit and reach test
Cardinale et al., 2003 [16]	16 women volleyball players, mean age: 23.5±4.6, WBV	VP = NR A = 10 mm F = 30–40–50 Hz a = NR	Duration: 60 s. Rest: 60 s. T: 240 s. Modality: half-squat position Position: knee angle 100° flexion	NA: sEMG (vastus lateralis) MP: NM
Giminiani et al., 2020 [56]	20 male sport science students, mean age: 22.7±0.6, WBV, RCT	VP = NR A = NR F = NR a = NR	Duration: 30 s. Rest: 240 s. Modality: half-squat position, heels raised Position: knee angle 90° flexion	NA: sEMG (vastus lateralis, biceps femoris, tibialis anterior, lateral gastrocnemius) MP: SJ
Marín et al., 2009 [51]	10 active males, mean age: 28.7±6.4, WBV, RCT	VP = vertical A = 2, 4 mm F = 30 Hz a = NR	Duration: 30 s. Rest: 60 s. T: 150 s. Modality: half-squat, basketball shoes Position: isometric half squat	NA: sEMG (vastus lateralis, gastrocnemius medialis) MP: NM

Note: CON = control, NR = Not reported, A = amplitude, F = frequency, a = acceleration, MVC = maximal voluntary contraction, sEMG = electromyography, WBV = whole-body vibration, CMJ = counter movement jump, SJ = squat jump, MIV = maximal isometric voluntary contraction, NM = not measured, NR = not reported, VP = vibrating platform, T = total WBV exposure time, s = second, mm = millimetres, NA = neuromuscular activation, MP = explosive power

### 3.3 Quality assessment and risk of bias

**Fig 2:**
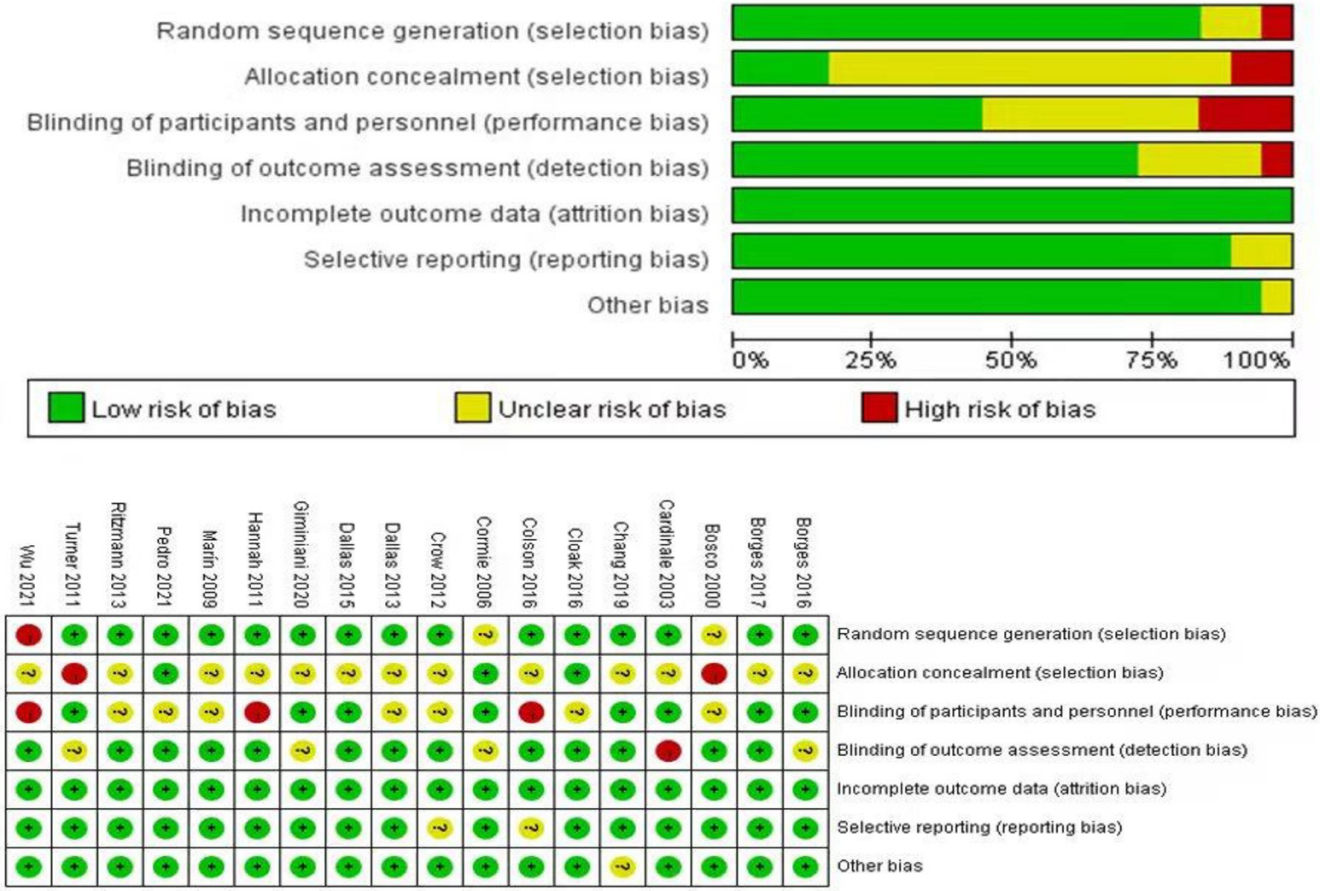
Methodological quality assessment of the included studies with Cochrane scale.

**Fig 3:**
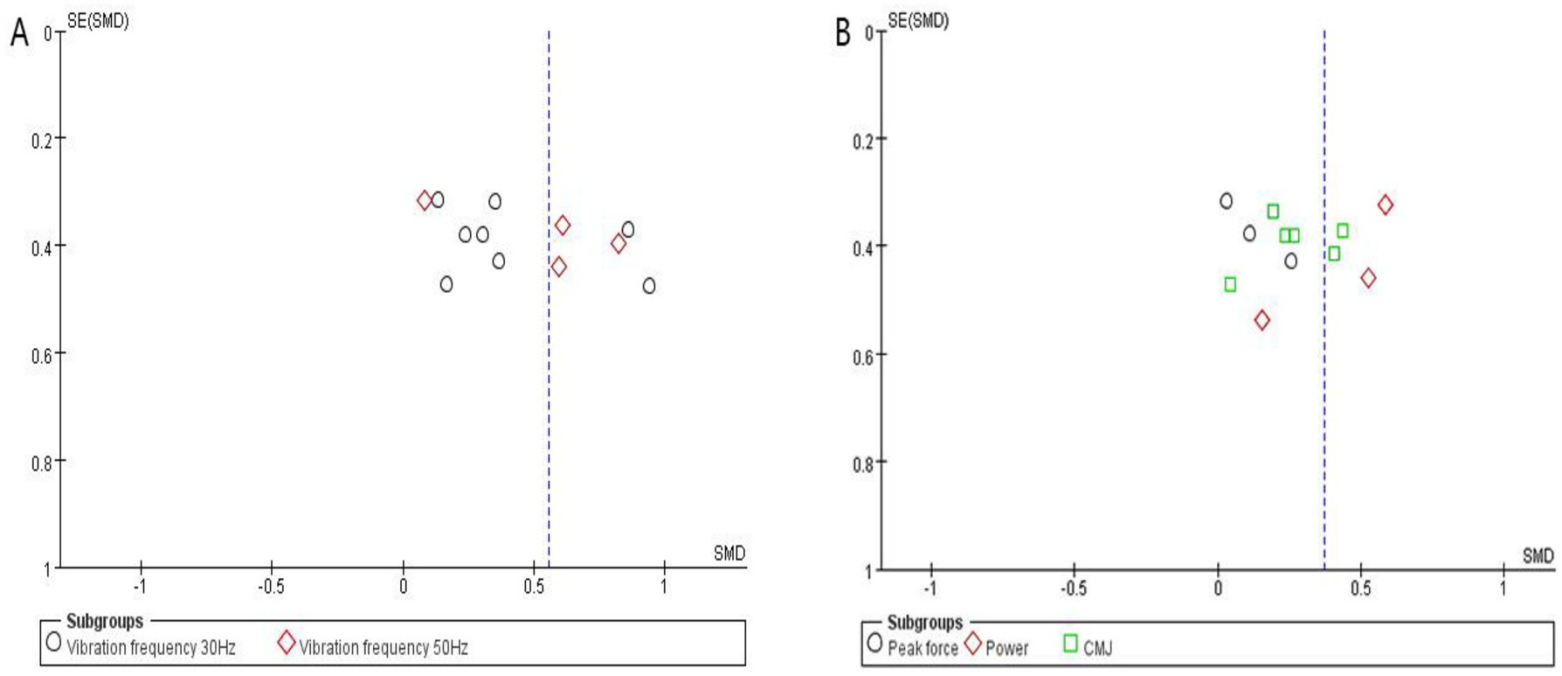
Funnel plots of included studies. (A) Neuromuscular activation post vibration versus previbration. (B) Explosive power post vibration versus previbration.

The Cochrane scores of the selected studies were determined (Fig 2). Thirteen studies showed unclear allocation concealment [16, 32, 53, 55–59, 61, 62, 64–66], two studies showed high risk [54, 60]. Seven studies showed unclear blinding of participants and personnel [51, 55, 58, 59, 63, 65, 66], and three studies showed high risk [53, 58, 61]. Publication bias was evaluated using the visual inspection of the funnel plot (Fig 3). The funnel plot was slightly asymmetric across neuromuscular activation and explosive power. These results suggested that marginal publication bias exists for neuromuscular activation, power, peak force and CMJ outcome studies.

### 3.4 Effects of WBV on neuromuscular activation

**Fig 4:**
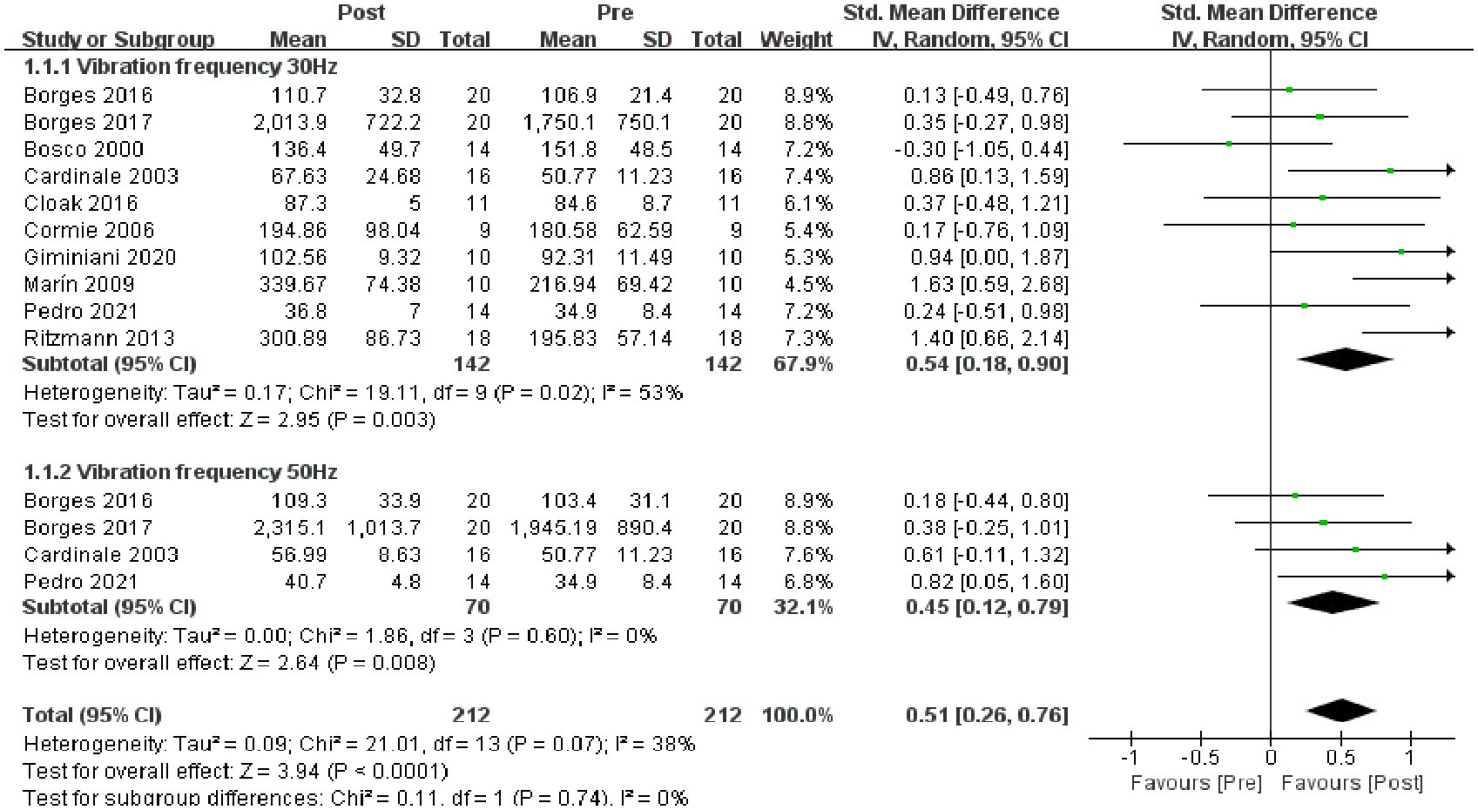
Meta-analysis of the acute effects of WBV on neuromuscular activation.

For neuromuscular activation (Fig 4), 10 studies were included in the meta-analysis. Meta-analysis showed a significant increase in neuromuscular activation (biceps femoris, quadriceps femoris, gastrocnemius and soleus muscles) after vibration than before vibration (SMD = 0.51; 95% CI: 0.26, 0.76; p<0.001), no significant heterogeneity was observed (I^2^ = 38%, p = 0.07) and strong evidence supported the positive effect of WBV training on neuromuscular activation. The data used for the meta-analysis of neuromuscular activation were the root-mean-square values of sEMG before and after vibration at 30 or 50 Hz.

Fig 4.1.1.1 shows that 10 studies with a vibration frequency of 30 Hz were included in the meta-analysis. Meta-analysis showed a significant increase in neuromuscular activation after vibration than before vibration (SMD = 0.54; 95% CI: 0.18, 0.90; p = 0.003), significant heterogeneity was observed (I2 = 53%, p = 0.02) and strong evidence supported the positive effect of WBV training on neuromuscular activation. Four studies with a vibration frequency of 50 Hz were included in the meta-analysis (Fig 4.1.1.2). Meta-analysis showed increased neuromuscular activation after vibration than before vibration (SMD = 0.45; 95% CI: 0.12, 0.79; p = 0.008), no heterogeneity was observed (I2 = 0%, p = 0.60), and evidence supported the positive effect of WBV on neuromuscular activation.

### 3.5 Effect of WBV on explosive power

**Fig 5:**
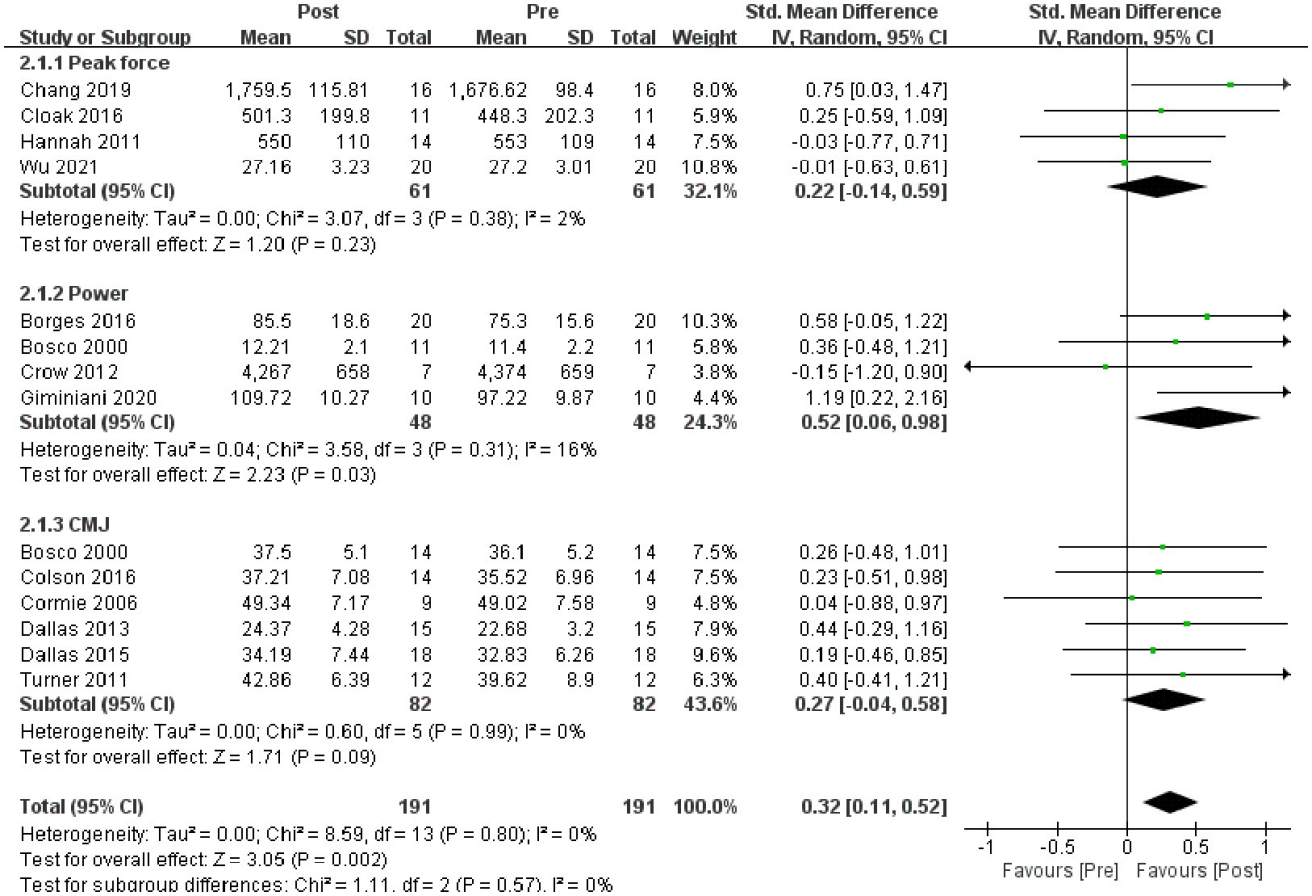
Meta-analysis of the acute effects of WBV on explosive power.

For the explosive power test (Fig 5), 13 studies were included in the meta-analysis. Meta-analysis showed a significant increase in explosive power after vibration than before vibration (SMD = 0.32; 95% CI: 0.11, 0.52; p = 0.002), no significant heterogeneity was observed (I^2^ = 0%, p = 0.80) and strong evidence supported the positive effect of WBV training on the explosive power. Meta-analysis explosive power is the data before and after the vibration frequency of 30 Hz.

For the peak force test (Fig 5.2.1.1), four studies were included in the meta-analysis. Meta-analysis showed no significant difference in peak force after vibration than before vibration (SMD = 0.22; 95% CI: −0.14, 0.59; p = 0.23), and no heterogeneity (I^2^ = 2%, p = 0.38). Thus, no evidence supported the positive effect of WBV training on peak force. Four studies with the power test were included in the meta-analysis (Fig 5.2.1.2). Meta-analysis showed increased power after vibration than before vibration (SMD = 0.52; 95% CI: 0.06, 0.98; p = 0.03), no heterogeneity was observed (I^2^ = 16%, p = 0.31) and strong evidence supported the positive effect of WBV training on power. For the CMJ test (Fig 5.2.1.3), six studies were included in meta-analysis. Meta-analysis showed no significant difference in CMJ after vibration than before vibration (SMD = 0.27; 95% CI: −0.04, 0.58; p = 0.09), no heterogeneity was observed (I^2^ = 0%, p = 0.99) and no evidence supported the positive effect of WBV training on CMJ.

## 4 Discussion

The aim of this systematic review was to evaluate the changes in neuromuscular activation through sEMG and explosive power after WBV exercise in athletes and training experience. The results suggested that WBV exercise may be a valid intervention to promote neuromuscular responses and increased muscle explosive power. Moreover, a strong level of evidence proved a positive effect of WBV training on the peak force and power test. By contrast, no change was noted in CMJ after analysing the included studies. Eight studies [51–54, 56, 57, 60, 61] evaluated healthy males, five studies assessed healthy women [16, 32, 58, 62, 63] and five studies assessed combined males and females [55, 59, 64–66]. The average methodological quality of this review was considered fair according to a Cochrane scale, and some items were not sufficiently documented in the included studies. Firstly, many studies on the Cochrane scale that were most rated as unsatisfactory were allocation concealment and blinding of participants and personnel. Secondly, two [60, 61] studies did not satisfy random sequence generation allocation concealment and blinding of participants and personnel. Studies with high risk were mainly lacking blinding participants and researchers, random sequence generation, and allocation concealment.

Previously, a systematic review showed that WBV exercises might promote desirable neuromuscular activation in different subjects [67, 68]. Three studies did not report improvement in muscular activity after WBV exercise [61, 69, 70], and this is in contrast to this study. Nine studies reported enhancing sEMG after WBV exercise [34, 57, 58, 62–64, 71–73], which also shows an increase in muscular activation after WBV exercise in different populations; this suggested that WBV training can improve neuromuscular activation. However, the contradiction may be because the studies employed different types, dissimilar biomechanical parameters of the mechanical vibration and positioning on the types. Whether shoes are worn also affects the transmission of stimulation and subsequent neuromuscular activation. One study showed that subjects wearing shoes had reduced neuromuscular responses to WBV exercise stimulation compared with those without shoes.

Meta-analysis showed that WBV exercises would lead to greater improvements muscle strength and power and CMJ [9]. Tibor et al.’s [47] meta-analysis of the acute effects and chronic effects of WBV on leg power in competitive and/or elite athletes found no significant effect, but the results of the present review suggested that the WBV would lead to acute effect on explosive power despite no effect on CMJ and peak force. The contradictory result may be caused by different exercise duration and vibration protocols, WBV parameters and subjects. The subjects of this meta-analysis, such as physical education students, sport science students, physically active men or women, recreationally active men, competitive and/or elite athletes were different. In addition, subjects received the vibration stimulus through non-standardized formats such as WBV platforms, cables and other vibrating devices. A sub analysis of the previous review compared the effects of WBV on performance in athletes versus sedentary subjects with CMJ as the main outcome, which may affect the analysis results [74]. The research protocol needs to be refined with further in-depth study on the effect of vibration training on CMJ.

Rhea et al.’s [75] systematic review suggested no clear evidence for the effects on muscular performance and power after short-term vibration in the same year. Nordlund et al.’s studies found no or only minor additional effects on power of WBV exercise. However, Alam et al.’s [67] systematic review and Dobbs et al.’s [76] meta-analysis suggested significant improvements in muscle power and strength after WBV exercise. The results of the present review suggested that the WBV would lead to increase muscle power and peak force. Cloak et al. [63] reported that acute WBV exercise elicited a positive neuromuscular activation amongst professional players and improvements in peak force, but benefits were not found in amateur players. The negative effect of WBV on peak force was also mentioned in a previous analytical review but was mostly observed in non-elite athletes [6]. The main reasons for the contradiction are the range of amplitude and frequency, the type of vibration and its application method, training protocol and subject characteristics. Therefore, WBV exercise can bring about improvement in muscle strength, power and peak force.

Fast-twitch fibres are more sensitive to vibration [77]. WBV exercises would increase explosive power because vibration stimulation can increase total stimulation intensity. Manimmanakorn et al.’s [72] review showed that WBV’s effect on jump performance was only about half of the effect size (0.59) observed in untrained healthy adults (0.96). Thus, the WBV effect decreases with increasing training status. That review reported effect sizes of 0.68 and 0.92 to WBV exposure shorter or longer than 10 min, respectively [72]. Insufficient WBV exposure time during specific skill practice hours can make the WBV effect trivial [78]. Moreover, none of the studies addressed whether the WBV effect could really exceed the duration of the session and increase athletic performance or its surrogate measures in minutes, hours or days after the initial exposure. Therefore, studying the length of vibration exposure is extremely important to improving explosive power and athletic performance.

## 5 Conclusion

The present review concludes that acute WBV training would lead to improvement in neuromuscular activation and explosive power in the lower limb, improving sports performance. However, the situation at other vibration frequencies remains unclear because only a vibration frequency of 30 and 50 Hz is analysed in this review. A strong level of evidence proves that acute WBV can improve CMJ performance, and this contradicts the results of this review. In addition, other types of exercise programs (e.g. resistance training) are recommended to improve sports performance. Further study protocol is needed to explore the possibility of finding a standardized protocol targeting sports performance in athletes, such as amplitude and frequency, type of vibration and method of application, training intensity and protocol, and characteristics of the subjects.

The present study has several limitations. Firstly, significant heterogeneity was found in sub groups of peak force [62] and neuromuscular activation [59], which may be attributable to factors, such as frequency or displacement, subject characteristics, body mass and footwear. After removing literature [59, 62], no heterogeneity remained because the study quality of literatures was high and without high risk, so they were not removed. Secondly, meta-analysis included participants with different characteristics (e.g. gender, fitness level) and involved comparison of outcomes in neuromuscular activation and explosive power. Thirdly, the effects of WBV on muscle strength and explosive power depended on the type of the vibration platform, frequency and amplitude [79]. This review failed to propose an optimal vibration parameter or exercise prescription due to the lack of consistency in the study methods. In addition, the method of neuromuscular activation assessment was based on sEMG of muscle contraction, and only sEMG was assessed in the meta-analysis for neuromuscular activation. Moreover, in the course of conducting this review, additional time was spent in seeking data from the authors of the original papers, and modifying the writing, which may have increased the period of our study. Future studies will continue to explore the effects of vibration training on neuromuscular activation and explosive power of the lower limb as high-quality studies increase.

## Supporting information

S1 Table Complete search strategy. (DOCX)

S2 Table PRISMA 2020 checklist. (DOCX)

S3 Table International prospective register of systematic reviews (CRD42021232984). (PDF)
