# Supplementary material for: Effect of whole-body vibration on neuromuscular activation and explosive power of lower limb: A systematic review and meta-analysis
Source: PLoS One. 2022 Dec 6;17(12):e0278637. doi: 10.1371/journal.pone.0278637 (PMC9725163; doi:10.1371/journal.pone.0278637)
Supplement: S1 Table — (DOCX) [file pone.0278637.s001.docx]

**Complete Search Strategy**

In the search strategy, a combination of terms was concluded: vibration, neuromuscular activation, and explosive power.

Databases: **PubMed, Web of Science, Google Scholar and EBSCO-MEDLINE**

**PubMed:**

*vibration:* (vibration*[MeSH Terms] OR vibration*[All Fields])

*neuromuscular activation:* ((neuromuscular activation*[MeSH Terms] OR neuromuscular activation*[All Fields]) OR (muscle activation*[MeSH Terms] OR muscle activation*[All Fields])

*explosive power:* (explosive power*[MeSH Terms] OR explosive power*[All Fields]) OR (muscle power*[MeSH Terms] OR muscle power*[All Fields]) OR (muscle strength *[MeSH Terms] OR muscle strength *[All Fields])

**Web of Science:**

*vibration:* TS = (vibration*[MeSH Terms] OR vibration*[All Fields])

*neuromuscular activation:* TS = ((neuromuscular activation*[MeSH Terms] OR neuromuscular activation*[All Fields]) OR (muscle activation*[MeSH Terms] OR muscle activation*[All Fields])

*explosive power:* TS = (explosive power*[MeSH Terms] OR explosive power*[All Fields]) OR (muscle power*[MeSH Terms] OR muscle power*[All Fields]) OR (muscle strength *[MeSH Terms] OR muscle strength *[All Fields])

**Google Scholar:**

*vibration:* vibration * OR whole-body vibration (WBV)

*neuromuscular activation: “*neuromuscular activation*”*OR“neuromuscular activation”OR “muscle activation”OR “muscle activation”

*explosive power: “*explosive power*”*OR “muscle power”OR “muscle strength”

**Embase:**

*vibration:* ‘vibration*’

*neuromuscular activation:* ‘neuromuscular activation*’ OR ‘muscle activation*’

*explosive power:* ‘explosive power*’ OR ‘muscle power*’ OR ‘muscle strength*’
