# Supplementary material for: Effect of whole-body vibration on neuromuscular activation and explosive power of lower limb: A systematic review and meta-analysis
Source: PLoS One. 2022 Dec 6;17(12):e0278637. doi: 10.1371/journal.pone.0278637 (PMC9725163; doi:10.1371/journal.pone.0278637)
Supplement: S3 Table — (PDF) [file pone.0278637.s003.pdf]

Effect of weight-bearing whole body vibration on neuromuscular activation and explosive power of human lower limb: a systematic review and Meta-analysis

1. Review question

PICO strategy, focus on Healthy competitive athletes or amateurs receiving WBV intervention (Intervention) control group (Comparison), all reported outcomes (Outcomes) were allowed if considered relevant to the studied population.

2. Searches

Web of Science, PubMed, EBSCO-MEDLINE and Google Scholar

3. URL to search strategy

Weight-bearing whole body vibration OR Resistance whole body vibration OR whole body vibration AND neuromuscular activation OR Neuromuscular OR Activation OR explosive power AND lower limb OR explosive power OR lower limb OR Lower limb neuromuscular activation AND lower limb explosive power OR neuromuscular activation AND explosive power AND lower limb. To ensure the accuracy of literature retrieval for relevant studies, the literature retrieval was completed with the assistance of librarians in Guangzhou Library. For the literatures in this review, two authors independently screened the title and abstract, followed by the full text. For the controversial literatures, the third author intervened in the examination.

4. Condition or domain being studied

Effect of weight-bearing vibration training on neuromuscular activation and sport performance

5. Participants/population

Literature inclusion criteria: subjects are healthy competitive athletes or amateur exercisers; Exclusion Criteria: Patients with cardiovascular disease, neuromuscular disease; cognitive, physiological dysfunction; spinal, upper and lower limb surgery within half a year; recent lower limb motor impairment activity.

6. Intervention(s), exposure(s)

(1) subjects randomly participate in the test conditions; (2) The study design is a comparison between vibration training, vibration with different negative weights and different vibration frequencies and a baseline or different test conditions; (3) Design protocol related to explosive power or strength training; Outcome Measures: Running, jumping, throw-related performance;(4) muscle activation is expressed by EMG activity indicators, explosive power is expressed by kinetic indicators, and the requirements are completed under weight-bearing whole-body vibration training conditions.

7. Types of study to be included

Randomized-controlled study, Randomized, crossover experimental study and experimental study.

8. Main outcome(s)

The aim of this systematic review is to use surface electromyographic or kinetic measures to assess the impact of neuromuscular responses or lower extremity muscle explosive power following exposure to WBV exercise in healthy competitive athletes or amateur sportsmen. Measures of effect: Surface electromyography, Dynamic indicators such as jump height.

9. Data extraction (selection and coding)

The review was conducted following four phases: (1) records were identified through database search and reference screening (Identification), (2) two reviewers (BB, AC) independently examined titles and abstracts and irrelevant studies were excluded based in eligibility criteria (Screening), (3) relevant full texts were analyzed for eligibility (Eligibility), and all relevant studies were included in the systematic review and (4) the disagreement was resolved by a third reviewer (EG). The same researchers were responsible for data extraction from the included studies. Data regarding study information (author and year), study design, demographics (sample size, age, sex, Body Mass Index), intervention protocols, WBV intervention and results were extracted.
Two reviewers used the Cochrane Collaboration tool to assess the risk of bias (BB, AC) and a third researcher was used to resolve conflicts (EG).

The level of evidence of the selected each selected publication was individually assessed by using The National Health and Medical Research Council hierarchy of evidence (NHMRC).

10. Risk of bias (quality) assessment

PEDro scale and The Cochrane Collaboration’s tool.

11. Strategy for data synthesis

All duplicates references were removed. The review was conducted following four phases: (1) records were identified through database search and reference screening (Identification), (2) two reviewers (BB, AC) independently examined titles and abstracts and irrelevant studies were excluded based in eligibility criteria (Screening), (3) relevant full texts were analyzed for eligibility (Eligibility), and all relevant studies were included in the systematic review and (4) the disagreement was resolved by a third reviewer (EG). The same researchers were responsible for data extraction from the included studies. Data regarding study information (author and year), study design, demographics (sample size, age, sex, body mass index), intervention protocols, WBV intervention and results were extracted.A total of 215 studies were identified through a database search and, after the removal of 45 duplicates, 170 studies were identified. During the screening process, 108 publications were excluded for not being related to

the research question, and the full text of 62 studies was reviewed in detail. After careful analysis, 48 studies were excluded (18 review articles, 16 did not use WBV and 14 books). Finally, 14 studies were included in the systematic review.
